# Health-related quality of life using EQ-5D among chronic myeloid leukaemia patients in health centres in Klang Valley, Malaysia

**DOI:** 10.1371/journal.pone.0256804

**Published:** 2021-08-27

**Authors:** Sharifa Ezat Wan Puteh, Azimatun Noor Aizuddin, Nor Rafeah Tumian, Jameela Sathar, Ellyana Mohamad Selamat

**Affiliations:** 1 Department of Community Health, Faculty of Medicine, Hospital Canselor Tunku Muhriz, Kuala Lumpur, Malaysia; 2 Haematology Department, Hospital Canselor Tuanku Muhriz, Kuala Lumpur, Malaysia; 3 Haematology Department, Hospital Ampang, Ministry of Health Malaysia, Ampang, Malaysia; International Medical University, MALAYSIA

## Abstract

Chronic Myeloid Leukaemia (CML) responds well with the targeted therapy drugs, Tyrosine Kinase Inhibitors (TKI), that give potentially long-term disease control for the patients. The objective of this study was to determine the disease burden and factors influencing the health-related quality of life (HRQoL) and health status of CML patients in Klang Valley, Malaysia. CML patients were recruited from haematological outpatient clinics in health centres in Klang Valley, Malaysia. A semi-guided self-administered questionnaire was used. HRQoL was measured by EQ-5D utility value and health status was by visual analogue score (VAS). Logistic regression analysis was conducted to determine the factors influencing HRQoL and health status. A total of 221 respondents participated, where more than half were Malay (56.6%), male (53.4%), and an Imatinib user (68.8%). Majority were diagnosed at the chronic phase (89.5%). The mean age of diagnosis was 41 years old. Significant determinant associated with HRQoL was age of diagnosis. These factors had no significant effect on the HRQoL of these patients regardless of types of TKI used and initial phase of CML. The overall HRQoL of CML patients were comparable to, if not higher, than the general population. Any TKI that was good enough to eliminate disease symptoms and erase patient’s worries, can possibly make CML patients have a better quality of life than typical cancer patients and even the general population.

## Introduction

Chronic myeloid leukaemia (CML) is a type of myeloproliferative neoplasm derived from myeloblast cells [1]. It is characterised by the presence of Philadelphia chromosome and its fusion gene, BCR-ABL1. The gene codes for an oncoprotein that stimulates the proliferation of myeloid cells. CML is the most uncommon of all the leukaemia, accounting for only 14% of overall leukaemia [2]. It affects all age groups, with the incidence rate varying between countries, from 0.3 to 1.6 for every 100,000 population [3, 4].

According to the Malaysian National Cancer Registry Report 2007–2011, the prevalence of CML in Malaysia was 573, that contributed to 12.5% of overall leukaemia cases in the country [5]. There is CML registry under the Malaysian Patient Assistance Program (MyPAP). MyPAP is a public-private partnership, between Ministry of Health Malaysia and pharmaceutical company Novartis, managed by a non-profit global health organization (The Max Foundation), with the support of Malaysian Society of Haematology. Most CML patients in Malaysia receive government funding assistance via MyPAP to gain access to therapy and optimal standard of treatment (tyrosine kinase inhibitors, TKIs) [6]. National Strategic Plan For Cancer Control Programme was introduced by the Ministry of Health Malaysia in 2016 to address the cancer care and management from a holistic view point that spans across primary prevention, screening, early detection, diagnosis, treatment, rehabilitation, palliative care as well as traditional and complementary medicine; and research [7].

Previous treatment options gave an average of 3 to 5 years of survival [1]. Providentially, current treatment options, TKI may offer a good long-term disease control [8–11]. Primary concerns of CML patients might include disease symptoms, treatment toxicity, effects of treatment and decision-making. They may also fear about their survival, loss of freedom, life disturbance and disability. Insecurity and emotional fatigue are among their concerns due to the potentially long-term course of illness, financial stress, increased risk of depressed mood and anxiety, conflict and family disturbance [12–15].

Quality of life (QoL) is an individual’s perception of their position in life in the context of the culture and value systems in which they live and in relation to their goals, expectations, standards and concerns [16]. When QoL is concerned with health and disease, it is usually referred to as health-related quality of life (HRQoL). HRQoL is a multi-dimensional term that is widely used to measure healthcare and public health intervention. It goes beyond indicators of public health, life expectancy and causes of death, and focuses on the effect on quality of life that health status had as well as measures of wellbeing and life satisfaction in relation to health status [17]. Living one year in perfect health may not be valued the same as living one year with disease; nevertheless, different patients may demonstrate different responses to a disease state. This is called utility (preference) value. HRQoL measures scales for utility values, using questionnaires such as SF-36, EuroQol EQ-5D and the World Health Organisation Quality of Life (WHOQOL). Quality-adjusted life years (QALY) estimates person-years lived at particular levels of health. It is typically measured on a scale of zero (death) to 1.0 (perfect health) by assigning various weights to potential health states. The usage of QALYs allows the incorporation of negative consequences of a treatment such as pain perception, and for conditions that are chronic but may have impact on length of life.

The objective of this study was to explore the determinants of HRQoL and health status of CML patients in Klang Valley, Malaysia. Klang Valley, that is conterminous with Greater Kuala Lumpur, has a current population of 10 million, representing about a third of the country’s total population [18]. By exploring the determinants of HRQoL and health status among CML patients, a better and improve holistic approach on the management of CML patients can be propositioned.

## Materials and methods

### Study design and study population

Between November 2019 and March 2020, CML patients were enrolled in this cross-sectional study involving two health centres in Klang Valley, Malaysia, one of which is the national centre of intervention for haematological cancers in Malaysia, that caters for most of the CML patients in the region. It is estimated that more than half of CML patients in the country are located here. CML’s targeted therapy TKI, although proven to be an excellent drug, is one of the most expensive outpatient drugs available, and the price is increasing every year [19, 20]. It put substantial pressure to the country’s health expenditure and healthcare delivery systems.

Eligibility criteria included: CML patients receiving treatment at the health centres and taking either Imatinib or Nilotinib but not both, for at least a month duration. Patients aged 18 and below must be accompanied with parents/carer when giving consent. The study was approved by the ethics committees of the participating centres as well as the Medical Research and Ethics Committee (MREC), Ministry of Health Malaysia (NMRR-19-1090-47137 IIR). All patients provided written informed consent in accordance with the Declaration of Helsinki.

### Study procedure and data collection

Hospital Ampang is a tertiary publicly funded specialist hospital located at the capital of Malaysia that has over 500 beds [21]. It caters for over 800 CML patients for at least 3 neighbourhood states, thus serving about half of the CML patients in the country. Hospital Ampang is the national centre of intervention for haematological conditions, in which its hematology expertise has become a National Reference Center by the Ministry of Health Malaysia [22]. It is the reference center for all hospitals of the Ministry of Health Malaysia (MOH) for cases of Hematology sub-specialty. It is also a leader in research for various diseases and treatments of Hematology at the national and international levels. Whereas Hospital Canselor Tunku Muhriz is one of the four public teaching hospitals in Malaysia that has over 1,000 beds [23]. It manages around 100 CML patients.

CML patients were recruited from haematological outpatient clinics in health centres in Klang Valley, Malaysia. Random sampling would then be done among the CML patients who came to the clinic. Patients were approached at the end of their medical consultation. Participation was voluntary and written informed consent were obtained from patients or the parents/legal caretakers of patients aged below 18 years. All eligible patients were informed about the purpose of the study and those consented to participate would be given a validated EQ-5D forms, in English or Malay language depending on their preference. Permission from EuroQol was obtained beforehand for the usage of EQ-5D forms for this study (registration ID: 30756). Patients were requested to complete the questionnaire after gaining their written consent, which would take about 5–10 minutes. Further information was gained from the hospital information system and patients’ medical notes.

EQ-5D is a standardised measurement tool for health status developed by the EuroQol Group to provide measure for health and quality of life in clinical and economic appraisals [24]. EQ-5D measures five dimensions, namely mobility, self-care, usual activities, pain/discomfort, and anxiety/depression [25] The questionnaire has been validated in Malay language [26, 27] with Cronbach’s alpha values of 0.58, and measurement had also been validated for the Malaysian population [28, 29] and it is the recommended patient-reported outcome measure tool by many health authorities such as the National Institute for Health and Care Excellence (NICE) in the UK [30]. There are two parts of the questionnaire, namely the EQ-5D for HRQoL and the EQ Visual Analogue Score (EQ-VAS) for the health status.

Each health state can be denoted using a five-digit number. The answers given for the five domains are then transformed to generate a summary score, which indicates the overall utility score. For EQ-5D-5L, the total possible health states are 3125 [31]. On the other hand, EQ-VAS records the respondent’s health on the same day on a vertical visual analogue scale from 0 to 100, in which 100 is labelled as ‘best imaginable health’ and 0 score is the ‘worst imaginable health state’ as rated by the respondents.

Patients characteristics collected include gender (male, female), ethnicity (Malay, Chinese, Indian, others), phase of CML on diagnosis [chronic phase (CP), accelerated phase (AP), blast phase (BP)], type of TKI used (Imatinib, Nilotinib), age of patients on diagnosis (years), duration of diagnosis (years), duration to start TKI (days), utility value and Visual Analogue Score (VAS). For continuous data such as age on diagnosis was categorised into <40 and ≥40 years old, duration of diagnosis (<6 and ≥6 years) and duration to start TKI (<95 and ≥95 days), all of which were based on the mean value that was used as the cut-off point between two groups.

Sample size calculated for the study was 200. It was determined using Cohen’s statistical power analysis with alpha 0.05, power 0.8 and Cohen’s D of 0.5.

### Data analysis

There were two categorical outcomes in this study, the QOL outcome measured by the EQ-5D-5L utility value score and the Health Status measured using EQ-VAS. The mean value scores of the two outcomes were measured and categorised into two groups by mean value of 0.89 and 81.96 respectively, as the cut-off point; high and low (HRQOL and Health status). Sociodemographic data, utility score and visual analogue score (VAS) were analysed using Microsoft Excel 2016 and Statistical Package for the Social Sciences (SPSS) software version 22 for descriptive, bivariate and binary multiple logistic regression analyses. A two-tailed p-value of <0.05 was considered as statistically significant.

## Results and discussion

### Characteristics of respondents

A total of 221 patients with response rate of 99.5% had consented and participated in this study, of which 56.6% were male and 53.4% were Malays. The phase of CML on diagnosis was predominantly in the chronic phase (89.6%), with the balance of 8.6% in accelerated phase and 1.8% in blast phase. More than half were Imatinib users (69%). Mean age of diagnosis (n = 221) was 40.87 ± 16.45 years old, with a range between 13 to 81 years old. Mean duration of diagnosis was 6.38 ± 4.65 years. Mean duration to start Tyrosine Kinase Inhibitor (TKI) medication was 94.24 ± 401.27 days. Table 1 illustrates the sociodemographic characteristics of the CML study population.

**Table 1:** Sociodemographic characteristics of CML study population.

	Variables	Frequency (%)	Mean (range)
1	Gender		
	• Male	125 (56.6%)	
	• Female	96 (43.4%)	
2	Ethnicity		
	• Malay	118 (53.4%)	
	• Chinese	72 (32.6%)	
	• Indian	29 (13.1%)	
	• Others	2 (0.9%)	
3	Phase of CML on diagnosis		
	• Chronic phase (CP)	198 (89.6%)	
	• Accelerated phase (AP)	19 (8.6%)	
	• Blast phase (BP)	4 (1.8%)	
4	Type of TKI used		
	• Imatinib	152 (68.8%)	
	• Nilotinib	69 (31.2%)	
5	Age on diagnosis (years)		40.9 (13–81)
6	Duration of diagnosis (years)		6.3 (0–23)
7	Duration to start TKI (days)		95.3 (0–1802)
8	Utility value		0.889 (0.116–1)
9	Visual Analogue Score (VAS)		81.96 (30–100)

### HRQoL by EQ-5D utility value

A total of 221 EQ-5D score sets have been analysed and converted into utility value. The mean score for the utility value was 0.89 (SD ± 0.14). A total of 76 of respondents (34.4%) reported 11111 in their health state which indicate full health and no problem in all the EQ-5D domains; mobility, self-care, usual activity, pain discomfort and anxiety/depression. A score set of 11111 will give a utility score of 1.0.

Table 2 shows CML risk factors towards HRQoL. Pearson’s Chi-square test was used to determine the relationship between the variables. Among CML patients, male patients had a statistically significant higher HRQoL (X^2^ = 5.62, p = 0.020) compared to female patients. Younger age on diagnosis had a statistically higher HRQoL (X^2^ = 7.50, p = 0.009) compared to those diagnosed at older age.

**Table 2:** Bivariate analysis of CML risk factors towards HRQoL utility value.

Independent variables	HRQoL Utility value		X^2^	P value	OR (95% CI)
	Low	High			
Gender					
• Male	32 (25.6)	93 (74.4)	5.62	0.020	0.50 (0.28–0.89)
• Female	39 (40.6)	57 (59.4)			
Ethnicity					
• Malay	40 (33.9)	78 (66.1)	0.36	0.567	1.19 (0.68–2.10)
• Non-Malay	31 (30.1)	72 (69.9)			
Phase of CML on diagnosis					
• Chronic phase	65 (32.8)	133 (67.2)	0.43	0.640	1.39 (0.52–3.68)
• Non-chronic phase	6 (26.1)	17 (73.9)			
Type of TKI used					
• Imatinib	49 (32.3)	103 (67.8)	0.00	1.000	1.02 (0.55–1.87)
• Nilotinib	22 (31.9)	47 (68.1)			
Age on diagnosis (years)					
• <40	27 (23.7)	87 (76.3)	7.50	0.009	0.45 (0.25–0.80)
• ≥40	43 (41.0)	62 (59.0)			
Duration of diagnosis (years)					
• <6	36 (32.4)	75 (67.6)	0.02	0.886	1.05 (0.59–1.84)
• ≥6	34 (31.5)	74 (68.5)			
Duration to start TKI (days)					
• <95	56 (30.9)	125 (69.1)	0.87	0.435	0.70 (0.34–1.48)
• ≥95	14 (38.9)	22 (61.1)			

* Pearson’s Chi-square test

Significant p-value < 0.05

As shown in Table 3, only age on diagnosis was significant as HRQoL determinant in the binary logistic regression model. Those diagnosed at a younger age has 2.2 higher incidence of high HRQoL [Adjusted OR 2.22, 95%CI (1.22, 4.05)] compared to those diagnosed at a later age. The goodness of fit of the model was assessed by Hosmer and Lemeshow test (p-value 0.249) and the model correctness was 67.3%.

**Table 3:** Binary logistic regression analysis for determinants of CML HRQoL by EQ-5D.

Factors	Wald	P value	Adjusted Odds Ratio (AOR)	95% CI
Gender				
• Male	3.573	0.059	1.783	0.979, 3.246
• Female				
Ethnicity				
• Malay	0.528	0.467	0.799	0.436, 1.464
• Non-Malay				
Phase of CML on diagnosis				
• Chronic phase	0.472	0.492	0.695	0.245, 1.966
• Non-chronic phase				
Type of TKI used				
• Imatinib	0.104	0.747	1.113	0.579, 2.139
• Nilotinib				
Age on diagnosis (years)				
• <40	6.803	0.009	2.223	1.220, 4.053
• ≥40				
Duration of diagnosis (years)				
• Recent	0.016	0.901	0.963	0.529, 1.751
• Former				
Duration to start TKI (days)				
• Early	0.533	0.465	1.342	0.609, 2.957
• Late				

Significant p-value < 0.05

### Health status by visual analogue score

The mean score for health status by EQ-VAS was 81.95 (SD ±15.0). Table 4 shows CML risk factor towards better health status. Pearson’s Chi-square test was used to determine the relationship between the variables. Younger age on diagnosis had a statistically better health status (X^2^ = 9.28, p = 0.003) compared to those diagnosed at older age. Table 5 shows the binary logistic regression analysis for determinants of CML health status by ED-VAS. Among CML patients, those diagnosed at a younger age has 2.2 higher incidence of better health status [Adjusted OR 2.23, 95%CI (1.276, 3.894)] compared to those diagnosed at a later age. The goodness of fit of the model was assessed by Hosmer and Lemeshow test (p-value 0.670) and the model correctness was 60.4%.

**Table 4:** Bivariate analysis of CML risk factors towards VAS health status.

Independent variables	VAS Health status		X^2^	P value	OR (95% CI)
	Low	High			
Gender					
• Male	64 (51.2)	61 (48.8)	0.11	0.787	1.09 (0.64–1.86)
• Female	47 (49.0)	49 (51.0)			
Ethnicity					
• Malay	56 (47.5)	62 (52.5)	0.78	0.419	0.79 (0.46–1.34)
• Non-Malay	55 (53.4)	48 (46.6)			
Phase of CML on diagnosis					
• Chronic phase	99 (50.0)	99 (50.0)	0.04	1.000	0.92 (0.39–2.18)
• Non-chronic phase	12 (52.2)	11 (47.8)			
Type of TKI used					
• Imatinib	79 (52.0)	73 (48.0)	0.60	0.470	1.25 (0.71–2.21)
• Nilotinib	32 (46.4)	37 (53.6)			
Age on diagnosis (years)					
• <40	46 (40.4)	68 (59.6)	9.28	0.003	0.43 (0.25–0.75)
• ≥40	64 (61.0)	41 (39.0)			
Duration of diagnosis (years)					
• <6	60 (54.1)	51 (45.9)	1.32	0.281	1.37 (0.80–2.32)
• ≥6	50 (46.3)	58 (53.7)			
Duration to start TKI (days)					
• <95	88 (48.6)	93 (51.4)	1.13	0.362	0.68 (0.33–1.39)
• ≥95	21 (58.3)	15 (41.7)			

* Pearson’s Chi-square test

Significant p-value < 0.05

**Table 5:** Binary logistic regression analysis for determinants of CML health status by visual analogue score.

Factors	Wald	P value	Adjusted Odds Ratio (AOR)	95% CI
Gender				
• Male	0.490	0.484	0.816	0.461, 1.443
• Female				
Ethnicity				
• Malay	0.980	0.322	1.328	0.757, 2.330
• Non-Malay				
Phase of CML on diagnosis				
• Chronic phase	0.082	0.774	1.143	0.458, 2.851
• Non-chronic phase				
Type of TKI used				
• Imatinib	0.333	0.564	0.837	0.457, 1.532
• Nilotinib				
Age on diagnosis (years)				
• <40	7.925	0.005	2.229	1.276, 3.894
• ≥40				
Duration of diagnosis (years)				
• Recent	1.442	0.230	0.710	0.406, 1.242
• Former				
Duration to start TKI (days)				
• Early	1.741	0.187	1.681	0.777, 3.633
• Late				

Significant p-value < 0.05

Chronic Myeloid Leukaemia (CML) is a type of blood cancer that is unique and differs from the rest of the leukaemia family. It is the most uncommon of all the leukaemia, accounting for only 14% of overall leukaemia [2]. Men are more affected than women [3, 4, 32]; a finding that was also found by our study. In fact, the male to female ratio was exactly the same (1.3:1) as findings from studies done at neighbouring countries, namely Singapore [33] and the Philippines [34]. The mean age of diagnosis was 40.9 years old, which was very similar to another local study by Kuan and Melaine Michael [32]. This figure is also similar to Thailand [35]. Thus, it is important to highlight that our findings represent the local and ASEAN region population well.

We found that Malays was the predominant ethnicity affected by CML, with more than half of CML patients were Malays (53.4%). A similar finding was found by Kuan and Melaine Michael [32] in which Malays had the highest rate, followed by Chinese. The same study also found that the majority of CML patients were diagnosed at the chronic phase (89.6%), which was very similar to ours (89.5%). Chronic phase (CP) is the initial clinical chronic phase of CML, in which the disease process is easily controlled, shows better response to treatment and has better prognosis [36]. This is followed by a transitional and unstable course, that is the accelerated phase (AP). The final phase, blast phase (BP), is more aggressive with a higher fatality rate. Among ASEAN countries, the majority of CML cases were diagnosed at the chronic phase (mean 84%, range 55–100%) [4]. The number of phases at diagnosis differs depending on the type of hospital the data collected. It may also depend on the type of services provided by the centres. For instance, a big and well-funded healthcare centre may have the facilities and can afford to diagnose and provide treatment to advanced stages of CML, thus it may have a higher number of advanced cases. Vice versa, small healthcare centres may refer most of the severe and advanced cases to tertiary centres prior to diagnosis.

We found that Imatinib was the main Tyrosine Kinase Inhibitor (TKI) used to treat CML, with more than two thirds (68.8%) of the patients using it. TKI is one of the most expensive outpatient drugs available, and the price is increasing every year [19, 20]. Funding issues are the main concern in the choice of medication used for CML worldwide, including Malaysia. Without any financial assistance, very few patients can afford any type of TKI, hence there are still low-income countries using other types of medication such as Interferon which has a poorer outcome to treat CML [3]. Imatinib, a first generation TKI, is cheaper than Nilotinib, which is a second generation TKI [37]. Thus, it is an expected finding to find Imatinib usage is higher than Nilotinib. Although Malaysia is blessed with governmental funding assistance for TKI under the Malaysian Patient Assistance Program (MyPAP) [32], nonetheless the allocation for TKI is not infinite. The allocation for the more expensive Nilotinib is understandably lesser compared to Imatinib.

Health-related quality of life (HRQoL) is a multi-dimensional term encompassing physical, mental, emotional and social functioning domains [38]. It is a measurable health outcome that incorporates patients’ functional capacity and well-being. HRQoL of cancer patients generally would be understandably lower than the general population. Cancer patients not only have to deal with the clinical progress of the disease and worsening of symptoms, but also the funding issues to deal with when spending for medication, treatment and long-term follow-ups. Their psychological states may be affected as well, as they may have to deal with the expectation of themselves, their family and the society. Moreover, their personal beliefs, social relationship and their relationship to the surroundings may also be of concern.

CML is truly a unique and novel type of cancer. It has a genetic component in which immature forms of myeloid cells arise to form an abnormal gene, BCR-ABL, that turns cells into CML cells that grow and reproduce out of control. Unlike other types of leukaemia, CML has targeted therapy drugs. Tyrosine Kinase Inhibitors (TKI) target directly at the BCR-ABL site gene hence ceasing the progress of CML. Hence nowadays, there is potentially long-term disease control or even a cure, as patients with CML who adhere to TKI therapy may live nearly full life spans. Therefore, when assessing the HRQoL for CML patients, one has to bear this in mind.

On a scale of 0–1.0, the higher utility scores may be interpreted as having achieved substantial quality in their life. Our study found that the overall mean of utility score for CML patients was very high (0.889). There was a very slight difference in the mean of utility score between Imatinib and Nilotinib users (0.886 vs. 0.893), and it was not statistically significant (p = 0.74). A study done by Azman, Sararaks [39] found that the health-related quality of life of the Malaysian general population was much lower at 0.650. Whereas for Visual Analogue Score, the scale is 0–100, with the higher score may be inferred as having achieved significant quality in life. We found that the mean for VAS among CML patients was 82, and the difference between the two TKI users was not statistically significant (p = 0.59). This was similar to a study done by Shafie, Hassali [27] that found the mean value of VAS in Malaysian general population was 82.2. However, another local study found the Malaysian general population to have a higher mean of VAS at 85.9 [26].

First of all, these findings showed that the HRQoL and health status among our CML patients were high. Chronic disease sufferers tend to have poorer health and more problems in their HRQoL [27]. However, this is not the case for our respondents. If having a score of 1 in the utility score is inferred as having 1 year of perfect health, then having a 0.9 utility score is considered quite up there. This may show that with TKI, the quality of life of CML patients was comparable, if not better, than the general population. This may also show that these patients were more appreciative of their life, that after undergoing the hardship of breaking bad news, dealing with the initial symptoms, then after the initiation of a ground-breaking medication like TKI, they were able to continue to function physically, mentally and socially. Therefore, for that they were more grateful of their life, hence having higher and better scores in HRQoL.

This is substantiated by our finding that different phases of CML on diagnosis did not show a statistically significant difference in terms of their utility score and VAS (p = 0.640 and p = 1.000, respectively). The later phases of CML, namely the accelerated and blast phases, typically have a more unforgiving and severe symptoms, yet their utility score and VAS were almost the same as those in earlier phase. Perhaps this is due to the elimination of symptoms by the TKI, making the CML patients having a better quality of life.

Imatinib and Nilotinib users did not show a statistically significant difference in terms of their utility score and VAS (p = 1.000 and p = 0.470, respectively). Despite many studies showing Nilotinib is a more superior TKI, has lesser side-effects with earlier and deeper molecular response [40–42], in terms of CML patients’ quality of life, our study found Nilotinib to be no different than Imatinib. Imatinib has been available in Malaysia since 2000, while Nilotinib was approved in the Ministry of Health Medical Formulary since 2012 [32]. Therefore, Imatinib has been around longer than Nilotinib. When a patient is compatible with a drug, such that it ables to eliminate disease symptoms and erase patient’s worry, it is unlikely for the clinician to change to a new medication, no matter how clinically superior the new medication is. Hence, regardless of the type of TKI used, it did not matter in the point of view of both patients and clinicians.

Our study found that the only significant determinants affecting HRQoL in CML patients was age of patients during diagnosis. Younger patients are more adaptive to changes, resilient to challenges and generally better at coping with problems. They are better at searching evidence and more IT savvy compared to older generation when looking for new information. Younger CML patients showed a better response to TKI treatment than older patients [43]. Older patients were associated with other comorbidity and polypharmacy hence were more at risk to have issues with treatment adherence than younger patients. Moreover, older CML patients were associated with more symptoms and signs as well as higher Sokal score at diagnosis than younger patients [44], therefore possibly affecting their quality of life.

The result of our study reflects the fundamentality of funding assistance for CML patients specifically, and for any cancer and chronic disease patients generally. The current pandemic era had inevitably affecting the economic and finances of people, it is providential that with the availability of the Malaysian Patient Assistance Program (MyPAP) helps to ensure appropriate treatments are accessible and affordable to patients.

## Conclusions

This study has demonstrated an assessment of HRQoL by EQ-5D and VAS among CML patients in Klang Valley, Malaysia. The overall HRQoL of CML patients was higher than the general population. Significant risk factors such as gender and age of diagnosis were associated with better HRQoL of the respondents. From this study, we found that regardless of the type of TKI used, nor which phase of CML the patients were initially diagnosed, these factors had no effect on the HRQoL of these patients. We deduced that any TKI was good enough to eliminate the disease symptoms and erase patient’s worries, thus making CML patients having a better quality of life than typical cancer patients and even the general population.
